# Applications of *In Ovo* Technique for the Optimal Development of the Gastrointestinal Tract and the Potential Influence on the Establishment of Its Microbiome in Poultry

**DOI:** 10.3389/fvets.2016.00063

**Published:** 2016-08-17

**Authors:** Stephanie M. Roto, Young Min Kwon, Steven C. Ricke

**Affiliations:** ^1^Department of Food Science, University of Arkansas, Fayetteville, AR, USA; ^2^Center for Food Safety, University of Arkansas, Fayetteville, AR, USA; ^3^Department of Poultry Science, University of Arkansas, Fayetteville, AR, USA; ^4^Cell and Molecular Biology Program, University of Arkansas, Fayetteville, AR, USA

**Keywords:** *in ovo*, poultry, gastrointestinal tract development, microbiome, supplements

## Abstract

As the current poultry production system stands, there is a period of time when newly hatched chicks are prevented from access to feed for approximately 48–72 h. Research has indicated that this delay in feeding may result in decreased growth performance when compared to chicks that are fed immediately post-hatch. To remedy this issue, *in ovo* methodology may be applied in order to supply the embryo with additional nutrients prior to hatching and those nutrients will continue to be utilized by the chick post-hatch during the fasting period. Furthermore, *in ovo* injection of various biologics have been researched based on the ability of not only supplying the chick embryo with additional nutrients that would promote improved growth but also compounds that may benefit the future health of the chicken host. Such compounds include various immunostimulants, live beneficial bacteria, prebiotics, and synbiotics. However, it is important to determine the site and age of the *in ovo* injection for the most productive effects. The primary focus of the current review is to address these two issues [the most effective site(s) and age(s) of *in ovo* injection] as well as provide the framework for the development of the gastrointestinal tract (GIT) of the chick embryo. Additionally, recent research suggests the colonization of the microbiota in the developing chick may occur during the late stages of embryogenesis. Therefore, we will also discuss the potentials of the *in ovo* injection method in establishing a healthy and diverse community of microorganisms to colonize the developing GIT that will provide both protection from pathogen invasion and improvement in growth performance to developing chicks.

## Introduction

Given the fact that the poultry industry has been a main contributor to the U.S. food supply over the past two decades, with an increase from $44.4 billion in value of production in 2013 to $48.3 billion in 2014, the necessity of capitalizing on the production of poultry is evident (1, 2). With the increasing trend to systematically remove sub-therapeutic levels of antibiotics from poultry feed due to public demand, poultry producers have been facing new challenges to compensate for lost product and profit *via* alternative routes (3, 4). For example, considerable research has been directed toward assessments on a wide range of potential feed additives, from essential oils to synbiotics, to make up for the negative effects on growth performance and immune system response observed in poultry with the withdrawal of antibiotic growth promoters from poultry feed (5–8).

There are two main approaches that may serve to aid in the optimization of broiler growth performance and health: the first one is related to epigenetics, and the second one to the gastrointestinal tract (GIT) microbiome. The role genetics has played in the improvement of broiler lines is apparent as summarized by Haventstein et al. (9). Genetic lines of broilers used in 1957 have more than tripled in size when compared to a genetic line used in 2001, from 1,009 to 4,402 g respectively [both genetic lines were fed modern-day diets; even greater differences in weight were observed in the birds fed diets respective of their time periods; (9)]. However, it is now believed that it requires an improvement at an additional level for optimized coordination of gene expression through epigenetic regulation to maximize the genitive potentials already carried in the genome of the broiler lines (10). Additionally, the current focus of breeding programs is being shifted toward improving feed conversion ratio rather than being concerned primarily with how to maximize the body weight observed in poultry due to the increasing cost for feed, which accounts for 70% of the total cost in poultry production (11). The second focus has been on the establishment and development of a healthy and mature GIT microbiome in poultry as it has been recognized for its critical role in the overall health and growth performance of poultry (12–14). An ideal route for optimizing bird performance and health *via* the GIT microbiome would be to establish a healthy and balanced GIT microbiome at the beginning of its formation rather than trying to alter an already established GIT microbiome. However, in order to attempt this challenge, it is essential to understand exactly when and how the GIT microbiomes are established during the early development of the birds.

Currently recognized as the most crucial time in the development of a young chick is the perinatal period (the last few days prior to hatch and the first few days after hatch); this time period is when intestinal development is occurring most rapidly (10, 15). The perinatal period is a transitional time in which the chicks undergo metabolic and physiological shifts from the utilization of egg nutrients to exogenous feed (10). However, with the current structure for the operation of hatcheries and the delivery of newly hatched chicks to broiler farms, the chicks are inevitably exposed to delayed feeding for 48–72 h. The starvation period introduced by poultry growers is brief yet occurring at a crucial time in their development causing stress on the young chicks, which may, in turn, lead to stunted GIT development (10).

In addition to the GIT development being hindered, the interference due to delayed feeding could be extending to the development of the GIT microbiome of these young chicks as well. For years, it has been known that *Salmonella* is able to pass vertically from mother to offspring *via* infected reproductive organs contaminating the yolk, albumen, or eggshell membranes of the newly forming eggs (16–20). With this in mind, more recent research investigating the microbiomes of various body regions in 1-day-old chicks *via* high-throughput sequencing of the 16S rRNA gene indicates an already diversely colonized GIT that can be detected in the cecal contents (21). This data may indicate that not only pathogenic bacteria but also beneficial or commensal bacteria are capable of being transferred from mother to offspring and may debunk the long held idea of eggs being sterile at the time of oviposition.

The *in ovo* method, which allows the delivery of various biologics and supplements to chicken embryos, may represent a means to both compensate for the starvation period that newly hatched chicks endure and facilitate early establishment of a healthy GIT microbiome before it is exposed to any pathogenic bacteria. Research regarding the *in ovo* method indicates its efficacy in supplying the young chick with critical biologics that can accelerate enteric development and digestion of nutrients (10). It has also been observed that early inoculation of a young chick with the native microbiota of a healthy adult bird can facilitate the development of an early GIT microbiome, thereby, leading to enhanced intestinal immunity as well as improved growth performance (22–26). Potentially, the newly developed GIT of an embryonic chick could be established by introducing a mixed culture of representative bacterial strains isolated from a healthy adult cecal microbiome.

The objective of this review is to serve as an overview for discussing the *in ovo* method as a possible approach to compensate for the repercussions of nutritional delays in early feeding on the GIT development. Additionally, this review will examine the potential of *in ovo* method in facilitating embryo GIT development directly by the introduction of probiotic cultures, indirectly *via* administration of prebiotics, or a combination of both in the form of synbiotics.

## Embryonic Gastrointestinal Tract Development and Activity

Chick embryo development has been well understood for decades; it has been utilized as a model system for investigations of embryonic development in other animals (27). A research article by Southwell (28) contains a more in-depth perspective on the anatomic intestinal differentiation in accordance with the embryonic staging of external features. Additionally, Roberts (29) authored a focused review on the molecular mechanisms and control in GIT development. The current section will serve as a brief overview of the development occurring from early stages of GIT development and differentiation up to the point of GIT function. However, because there is variation observed among individual chick embryos in their developmental stages in relation to age (28), this process will be discussed in sequential order without referring to specific stages in this review. This section contains general information regarding the GIT development of intestinal organ and epithelial differentiation and the initial activity observed in GIT, with information pertinent for the understanding of the *in ovo* method. Multiple outside sources (peer-reviewed, scientific journal articles, and academic textbooks) discuss chick embryology to a much greater extent (29–34).

During gastrulation, the foundation of the alimentary tract is initially observed at approximately 18 h post-oviposition (35). The alimentary tract stems from a band of cells indicating cranio-caudal orientation of the future embryo, recognized as the primitive streak [(35); the research conducted by Conrad Hal Waddinton in the 1930s identified the hypoblast as the inducer of the primitive streak and acquired information regarding the amniote organizer: Hensen’s node. Waddington’s experimental research and inductive action findings in embryology have been evaluated in a comprehensive review, authored by 0 (27)]. The function of the primitive streak is to serve as a route for cells to be brought in from the outer embryo layer and become the mesoderm and endoderm for the newly forming embryo (36). The alimentary tract begins as a cylindrical tube with inner and outer layers, the endodermal and mesenchymal layers, respectively (37). The morphogenesis of the alimentary tract gives rise to the digestive system, among other organs and systems (38). The foregut arises as individual cells originating from the epiblast (region at the surface of the area pellucida), migrate through Hensen’s node, and remain anterior to Hensen’s node [this occurs at approximately 24 h post-oviposition (39, 40)]. Regression of the primitive streak begins with Hensen’s node moving to a more posterior position forming the anal region (40).

The formation of the head fold from the primitive streak allows for the formation of the foregut. The foregut lengthens toward the posterior end of the embryo and ends at the anterior intestinal portal [not a closed end, but an opening between the foregut and the midgut (32)]. Organogenesis from the foregut eventually gives rise to the organs and glands beginning at the pharynx, moving posterior along the GIT (41). The developments from the pharyngeal region are the esophageal region, crop, spleen, stomach, proventriculus, gizzard, liver, gallbladder, pancreas, intestines, and ceca (32, 42).

The formation of the hindgut extends posterior, beginning at the posterior intestinal portal and occurs similarly to that of the foregut (32). The hindgut differentiates into the posterior organs: the cloaca, allantois, and the bursa of Fabricius (32). The development of the hindgut is delayed when compared to that of the anterior regions and foregut. The midgut is distinguishable by its lack of ventral limits; it is open to the yolk sac. Eventually, the midgut is only the yolk sac stalk (32).

As time progresses (approximately 4–9 days post-oviposition), the morphogenesis of each intestinal organ and gland occur. The smooth endodermal layer forming the lumen of the intestine changes shape, from circular to an elongated ellipse and finally to a triangular shape. The triangular shape gives rise to longitudinal ridges, termed previllous ridges, which increase in number until eventually becoming villi lining the intestinal endoderm (31). The hypothesized mechanism by which these ridges and villi form is due to a combination of inward buckling of the endodermal layer as the outer smooth muscle layers differentiate and cell proliferation in both the mesenchyme and endoderm layers occur (37).

In addition to the development and height of villi prior to hatch, enzymatic activity is of particular interest in that several enzymes are integral in nutrient digestion for the young chick [for example sucrose-isomaltase (SI), lipase, and sodium-glucose transporter-1 (SGLT-1)]. Uni et al. (43) conducted experiments to observe pre-hatch villi and enzyme activities of the small intestine. At day 15 of incubation [preparation for emergence stage, Ref. (44)], rudimentary villi were observed and, by day 17, they were displaying differing stages of development. At day 20 of incubation, the villi could be visualized in three different stages, with varying maturity levels. The most mature villi were elongated pear-shaped, less mature villi were shorter, and the most nascent villi were branching at the base of the mature villi (43). Because chickens are precocial, it is essential that enzymes are active, even minimally, prior to hatch, which is the case for SI, SGLT-1, and lipase expression (43, 45). Both SGLT-1 and SI activity during incubation on days 15 and 17 were low; yet, on day 19, activity was heightened before any exogenous carbohydrates had been ingested. In summary, the villi along with enzyme activity are developing and active prior to hatch, preparing the young chick for the transition to nutrients from exogenous nutritional sources.

Enterocytes in the developing intestine are small, circular, and non-polar with no defined brush-border membrane. Immature enterocytes proliferate along the villi, as there are no crypt cells present in the small intestine in the late embryonic stages; at hatch, only a single crypt per villus has been observed (46, 47). This is in contrast to mature enterocytes, which are polar and exhibit a defined cellular structure allowing for optimal absorption (48). Mature avian intestinal epithelial cells are continuously being regenerated by proliferating crypt cells migrating up toward the apical surface of the villi and differentiating into enterocytes during transit (46). The transitional period of the first days post-hatch indicate that there is no defined areas of enterocyte proliferation; some occurs in the immature crypts, while some continue to occur along the villi (46). Full development of various regions of the chicken embryo, more specifically the GIT, can be more thoroughly reviewed in additional literature sources (32, 41, 49).

The mucosal layer of the intestine plays roles in the protection of the epithelial lining as well as transportation of materials between the lumen and the brush-border membrane (43). The intestinal tract has varying mucosal structures according to region (50). Development of mucus-secreting cells is initiated in the last stage of embryonic development; at this stage, these cells contain only acidic mucin (43, 51). The acidic mucins, although not entirely understood, may serve as an innate barrier to ward off bacteria, as the acquired immune system is not yet functional (52, 53). This is contrary to mature goblet cells, which contain both acidic and neutral mucins. Additionally, the number of goblet cells in the mature small intestine is evenly distributed and is increased in proportion to the enterocytes. However, in chicken embryos, goblet cells increase in number from the duodenum to the ileum (43).

**Figure 1:**
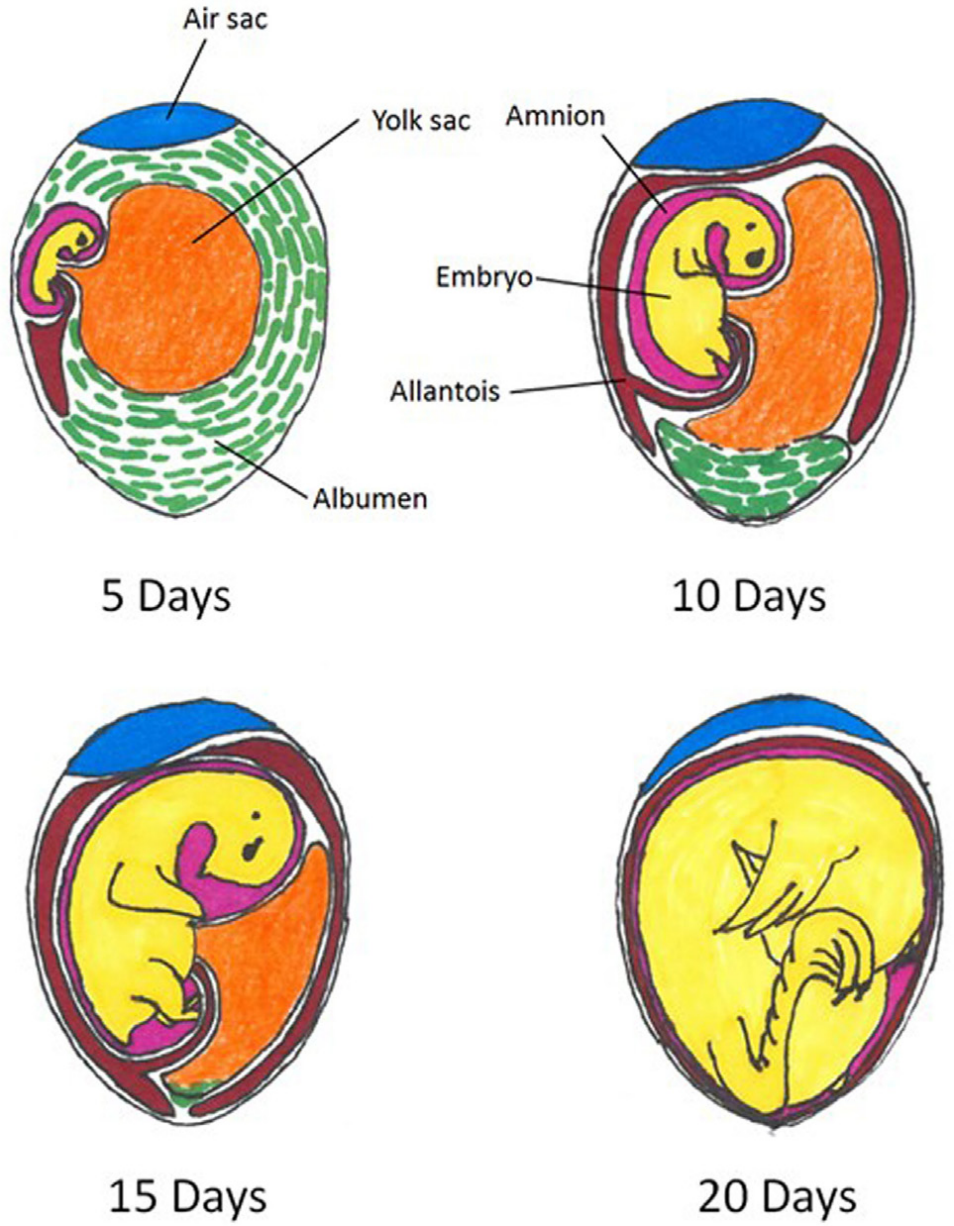
**Spatial relations within an embryonated chicken egg at 5, 10, 15, and 20 days of incubation**. Colors indicate differing compartments: embryo = yellow; air sac = blue; amnion = pink; allantois = red; albumen = green. Figure adapted from: A. L. Romanoff, Cornell Rural School Leaflet, September, 1939.

The spatial relations of the embryonated egg and extra-embryonic structures at various days of incubation are depicted in Figure 1. This pictorial representation of the embryonated egg organization can be referred to throughout the remainder of the current review as *in ovo* methodology is discussed.

## Importance of Early Feeding

The delayed feeding is known to have significant impacts on the future development of various systems in a chicken. Avian species with rapid early development of the intestine and liver have been correlated with high growth rates overall (54). Studies have revealed connections of early feeding not only to the overall performance but also specifically to intestinal, muscle, and immunological development and yolk-reserve utilization (55–57). Early feeding allows the small intestine to continue its growth and development rapidly because, at the time of hatch, the GIT is not yet fully developed (45, 58). Increasing villi height and crypt depth allows for increasing absorption and digestive capabilities (59–63). The transition from the lipid-rich yolk contents to carbohydrate- and protein-rich exogenous nutrient source is only possible by the appropriate development of the GIT (62, 63). Sklan and Noy (64) reported that fasting of chicks slows the passage of the yolk contents through the yolk stalk to the intestines. Potentially, it is the absence of feed that results in the lack of peristaltic movement or the negative pressure in the abdominal cavity to facilitate the passage of intestinal contents (65, 66).

When all the reserves from the yolk have been used post-hatch, the chick depends on the availability of exogenous nutrients for digestion and absorption *via* the GIT for continued growth and development. It is critical that GIT development be maintained and completed within the first few days post-hatch (67, 68). Studies have shown that the growth of the GIT post-hatch exceeds the rest of the body by fivefold (58, 69). There is a direct relationship between intestinal weight gain and secretory enzymatic activity (59, 70). This provides support for the necessity of chicks to gain access to external feed at the earliest possible time to continue GIT growth and development.

Intestinal cell proliferation is necessary to initiate replacement of embryonic enterocytes; this is stimulated by feed intake. These mature enterocytes allow excretion of digestive enzymes that is essential for absorbing external nutrients. This entire process may take up to 2 weeks (71); intuitively, delay in external feed will delay the process of the replacement of enterocytes. Sklan (63) demonstrated varying developmental intervals of villi height and growth among different regions of the intestine post-hatch. In the first 4 post-hatch days, the villi height is increased by 50% (60, 72). Maturation of the GIT is continued by exogenous feed intake, which increases villi length and enzymatic activity of the small intestine (68).

The delay in feeding causes chicks to enter starvation mode; the reserves intended for muscle protein are being mobilized to continue gluconeogenesis, while the newly hatched chick gains the capability of digesting exogenous glucose sources (68). The chick allocates the limited reserves to the upkeep of thermal regulation and metabolism, which restricts growth and development (73, 74). Uni and Ferket (55) characterized these shortages as the “hatchability quality” phenomena in which hatchlings are unable to endure the transitional periods before, during, and after the “starvation” phase. Those that do survive are often times underweight with poor meat yield, ineffective in utilizing feed properly, and more prone to disease.

Although some experiments demonstrated that the development of the GIT and other systems in delayed fed chicks can catch up within 1 week to the levels of immediately fed chicks, the long-term effects of the delayed fed chicks are evident. Several researchers have shown that the initial lag in development from delayed access to feed is perpetuated through to market age (75–78). Nir and Levanon (79) concluded that the amount of time chicks were held without access to food, 24 and 48 h, delayed the time it took to reach market weight by 1 and 2 days, respectively.

Ferket (10) proposed that the first meal may dictate which genes in the young chick become activated. For example, chicks enduring a starvation period may have different gene expression patterns for potentially the remainder of their lives when compared to chicks that are immediately fed post-hatch (10, 80). Nutritional imprinting suggests the ability of establishing desired traits to embryos in the egg or newly hatched chicks. It is speculated that the effects could be numerous: tolerance to various stressors (immunological and environmental), energy utilization, and caloric efficiency. Angel and Ashwell (81) demonstrated the long-term effects of conditioning with minerals in early feed. The data obtained compared chicks (0–4 days old) fed with a control diet (0.50% available phosphorus; NRC levels) with chicks conditioned with restricted levels of phosphorus (0.25%). The data indicated that, when fed finisher diets deficient in phosphorus, the birds conditioned with lower levels of phosphorus exhibited improved body weight gain and feed conversion at 38 days of age when compared to those fed control diets (81). Yan et al. (82) observed similar trends: ileal absorption was increased for phosphorus and calcium in broilers conditioned with slightly deficient diets from hatch to 18 days when compared to those fed control diets. Overall, it is not only what is fed to young chicks but also the time in which they are fed (perinatal period) that appears to have the greatest influence on how the birds may react to environmental conditions later in life (10, 80).

## GIT Microbiome Development

The assumption of chickens being hatched germ-free was prevalent when methods to characterize the poultry microbiome were strictly culture-based. However, with advancements in culture-independent methods, it is becoming more apparent that this conventional perception is no longer valid (83). To some extent, this should not be a surprise as it was already established that eggs may be contaminated at the time of lay either by vertical or horizontal transmission (84). Bacterial spoilage of eggs has been recognized for years, but it has also been shown that those microorganisms causing spoilage have certain capabilities allowing them to contaminate the egg and overcome its defenses (85). Among these, the contaminating microorganisms must contain enzymes and mechanisms to break down egg protein into nitrogen and carbon sources, and possess the ability to avoid inhibiting components found in the egg albumin, such as conalbumin, lysozymes, ovotransferrin, and ovomucoid (85–87). A fairly diverse group of microorganisms have been observed as contaminants associated with egg surfaces and their contents (88–90).

Data collected from Deeming (91) suggested that microorganisms in the yolk sac of the embryos of various avian species to be a fairly commonplace occurrence in healthy birds. These data refute the idea of avian eggs and their embryos being sterile before hatch since the embryo internalizes the yolk in the late stage of development (83, 91). Consequently, the notion that the intestinal microbiome of the chick is acquired on the day of hatch, thereafter, appears to be incorrect (92). More recent research indicates that the colonization of the intestinal tract may occur prior to the time of hatch by the prevalence and diversity of bacteria observed in 1-day-old chicks’ cecal contents by deep sequencing of the 16S rRNA gene (21). Additionally, diverse microbial populations have been identified in the intestines of chick embryos as early as 16 days of incubation using molecular and microscopic techniques (83). It seems plausible that both vertical and horizontal transmission of bacteria may occur. Because microorganisms from the hatching environment as well as from the mother are able to penetrate the eggshell and reach the yolk and albumen, it is likely these microorganisms go on to colonize the embryo intestinal tract as the yolk is imbibed (93).

Two independent studies used culture methods in identifying that chicken embryos were colonized with bacteria from samples taken at the end stage (17th to 20th days of incubation) of embryo development (94, 95). Kizerwetter-Świda and Binek (95) reported findings of *Enterococcus* sp. as the most frequently observed species among all the sampling ages, with the newly hatched chicks displaying the most complex microbiota, followed by the samples taken from 20 days chick embryos. It was also determined that the ceca had the highest bacterial counts, followed by the yolk sac. Binek et al. (94) reported results revealing the identification of similar microbial populations: *Entercoccus* sp., *Micrococcus* sp., and *Bacillus* sp. Additionally, Ilina et al. (96) used terminal restriction fragment length polymorphism (T-RFLP) to determine the structure and composition of the microbial populations present in the GITs of chick embryos on the 16th day of incubation in two different breeds of Hajseks chickens (Brown and White breeds). This study indicated that by 16 days of incubation, the chick embryo contains microbiota with relatively rich taxonomic diversity, reporting that the Hajseks Brown breed samples to contained 38 different phylotypes, while 30 were observed in the Hajseks White breed samples (96).

## *In ovo* Administration

Early in the 1980s, *in ovo* vaccination against Marek’s disease (MD) was established as a reliable method to ward off the infection due to exposure to the virus (97). Prior to *in ovo* injection, the MD vaccine was distributed post-hatch; however, vaccinated flocks occasionally continued to experience extensive mortality due to the MD virus. One of the potential factors attributing to the loss observed was that the post-hatch vaccinated birds were exposed to MD prematurely, allowing insufficient time for the young chicks to build immunity to the vaccine (97). Sharma and Burmester (97), recognizing the ability of late-stage embryos and fetuses to support immune responses to viral and bacterial antigens (98, 99), used the *in ovo* injection for the MD vaccine in embryonic chickens. They observed significantly greater protective indices when vaccinated at embryo stage (regardless of day of *in ovo* injection between the 16th to 20th days of incubation; vaccination at the 18th day revealed the greatest protection) compared to those vaccinated at hatch (*p* < 0.05), while having no effect on hatchability (97).

Because of the success with *in ovo* vaccination, extensive experimentation has been conducted with the injections of various biologics, such as nutrient supplementation, hormones, and immunostimulants. However, skepticism of the technique was also raised based on lack of optimization in deliverance (age, volume, location of injection, as well as other factors), stress caused to the embryo by disruption of the internal environment or osmotic balance, and insufficient evaluation for the optimal individual or mixed substances for injection or their appropriate concentrations for delivery. There are several factors that may impact the method of delivery, for example the most suitable site of injection (amnion, allantoic cavity, yolk sac, and air sac) might be impacted by the chemical and physical characteristics of the injectable solution (100, 101). As the logistical issues were identified, poultry researchers, realizing a commercial opportunity associated with this novel delivery approach, have performed various experiments to address the aforementioned issues.

Since the initial introduction of *in ovo* technique, there have been numerous patents for automated deliverance with variations in site of injection, solution injected, age of injection, and method of automation (102–106). Among the various patents for automated injections, Uni and Ferket (107) patented a method for the delivery of *in ovo* injections that has come to be widely accepted among investigating researchers in terms of the age, location of injection, volume of injection, and a validated array of biologics that may be injected (107).

## Sites of *In ovo* Injection

**Figure 2:**
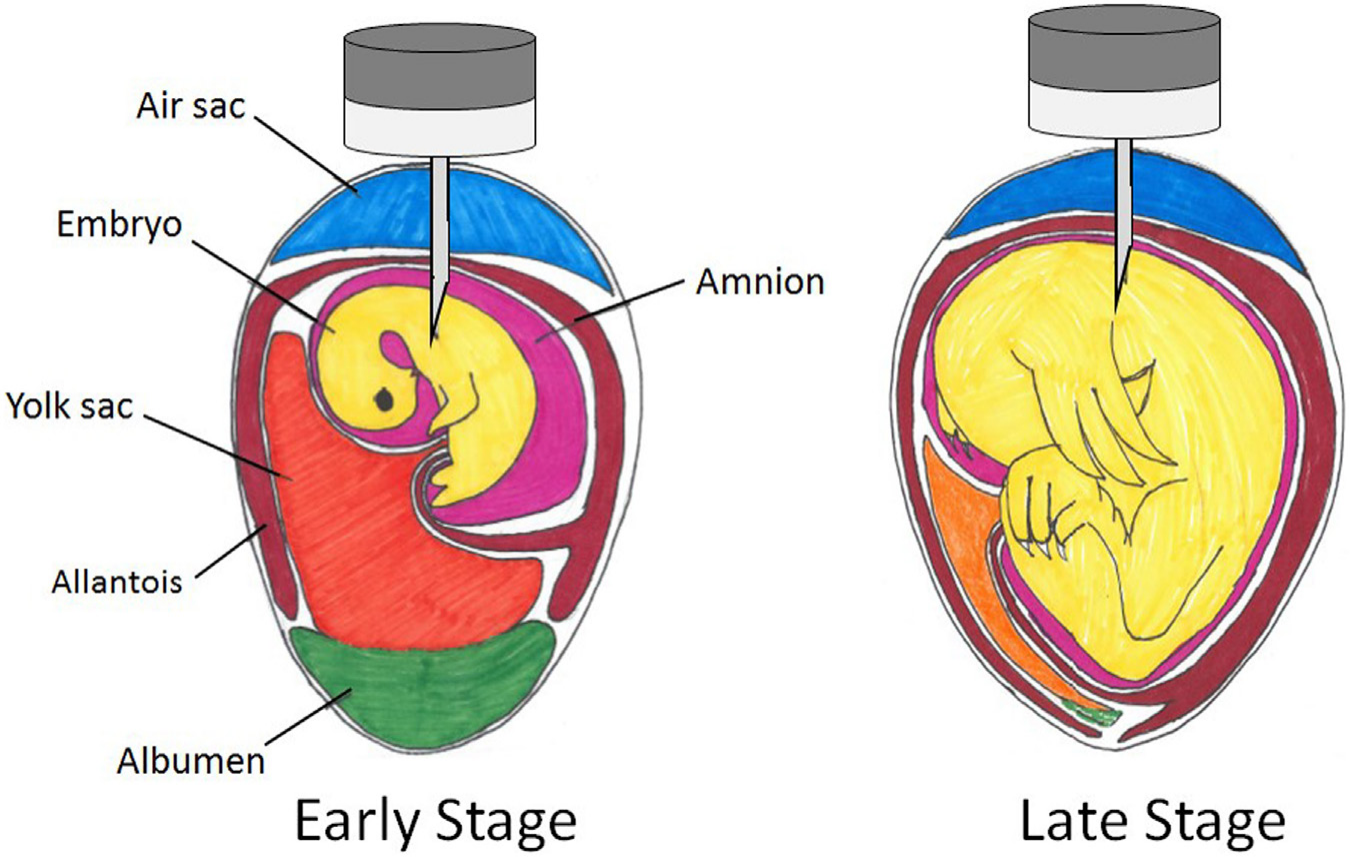
**Spatial relations within an embryonated chicken egg at early and late stages of incubation with possible *in ovo* injection sites**.

In the late stage of embryonic development, there are five regions through which an *in ovo* injection may be delivered: the air cell, the allantoic membrane, the amniotic fluid, the yolk, and the embryo body [Figure 1; Ref. (108)]. The patent Uni and Ferket (107) developed states that the ideal time period for injection was late-term avian embryo with delivery to the amniotic fluid. The embryo consumes the amniotic fluid and its contents are exposed to the intestines and the enteric cells that comprise them. Therefore, substances administered to this region will be consumed along with the amniotic fluid and presented to enteric tissues (107). Wakenell et al. (108) evaluated the consequences of *in ovo* injection of the MD vaccine to eggs on the 17th and 18th days of incubation at various locations. The results indicated that the needle should pass through the air cell and the allantoic fluid in order to inject and dispense the vaccine to either the amniotic fluid or the embryo body to achieve the greatest protection efficacy (Figure 2). This deliverance resulted in over 90% protection regardless of day of vaccination, while injection of vaccine into either the air cell or allantoic fluid resulted in less than 50% protection (108). The precision in the depth of the injection is crucial; the needle not being deep enough into the egg will result in the dispersion of the vaccine to the air cell or allantoic fluid (<50% protection), while injecting the needle too deep may cause trauma to the embryo [Figure 2; (108, 109)]. However, Islam et al. (110) obtained differing results, indicating poor response to vaccination when delivered to the extra-embryonic fluid. As Wakenell et al. (108) indicated, the reasoning for the differing results between the studies may be based in the “extra-embryonic” fluid not being differentiated into compartments. Therefore, the injections dispersed to the air cell, the allantoic fluid, and the amniotic fluid were all considered the same region: extra-embryonic.

Since the *in ovo* technique had become established, it continues to be further examined; more recent research involving *in ovo* techniques follows the age, location, and volume of injection recommended in the Uni and Ferket (107) patent [hereafter, studies discussed followed the recommended location and age as given by Uni and Ferket (107); those differing from the Uni and Ferket (107) patent will be stated as such]. The variable in the majority of *in ovo* studies have focused on what supplement is injected, such as nutrients, hormones, immunostimulants, or other biologics, attempting to promote growth and stimulate the immune system. As noted by Donaldson (111), adding glucose to water for chicks was not only ineffective but was detrimental as it suppressed gluconeogenesis enzymatic activity. Since different supplements exhibit different interactions, the effectiveness of each prospect needs to be assessed on an individual basis. The sections that follow will review the various biologics used in *in ovo* injections.

## Nutrient Supplements: Macromolecules, Amino Acids, Vitamins, and Minerals

A major intention of pre-hatch feeding is to equip the embryo with the nutrients necessary to continue intestinal development post-hatch at or close to the same rate as pre-hatch. Supplying the embryo with exogenous nutrients would allow the GIT to develop the structures and functionality to properly digest and absorb nutrients immediately when exogenous nutritional supplementation is provided after hatch (107). These nutrients, along with the yolk sac reserves, can contribute not only to maintaining the systems and metabolism already established but also to continuing growth, development, and proper nutritional status (112). Numerous studies have been conducted investigating the efficacy of *in ovo* injection of various biologics in poultry, including nutrient supplements (Table 1).

**Table 1:** **Summary of the effects studied regarding *in ovo* injections in chicken embryos at various locations and times of incubation and biologics supplemented**.

Biologics injected	Reference	Stage of incubation^a^	Location of injection	Results
Carbohydrates	(51, 66, 113–115)	Late stage	Amniotic fluid	Trophic effects on small intestine and effects on goblet cell activity; effects on embryonic metabolism and body weight
Amino acids	(116–118)	Late and early stage	Amniotic fluid, yolk sac, air cell, site not specified (needle length and narrow/broad end of egg given)	Effects on chick-to-egg ratio, body weight, bursal weight, and thymus weight; effects on body weight in relation to location and day of injection; effects feed intake, feed conversation ratio, and immune response
Hormones	(119–121)	Late and early stage	Albumen	Effect on muscle content; effects on body weight, skeletal growth, feed efficiencies, and adipose tissue development
Prebiotics, probiotics, synbiotics	(122–125)	Early stage	Air cell, amnion	Effects on muscle fibers and histology; effects on *Salmonella* colonization; effect on final body weight gain and pancreatic enzyme activity; effect on number of bifidobacteria in feces
Proteins (antibodies)	(126, 127)	Late and early stage	Yolk sac, albumen, amniotic fluid	Effects on body weight and muscle mass varied among injection locations; effects on antibiotic residue detection
Immunostimulants	(128–130)	Late stage	Amniotic fluid	Effects on *in vitro* bactericidal activity of heterophils and protection against *Salmonella* invasion; effect on macrophage and antibody response

*^a^Day of incubation, early stage = 0–12 days of incubation; late stage = 13–21 days of incubation; see reference for exact day of incubation*.

Feeding of carbohydrates (51, 113, 114), proteins and amino acids (116, 117), vitamins (66, 131), or other modulators (119, 132) through *in ovo* injection have been evaluated. When Smirnov et al. (51) inoculated eggs with carbohydrates (maltose, sucrose, and dextrose), the results showed that the additional energy source enhanced the development of goblet cells and increased the villi surface area in the intestines. The same carbohydrate mixture was applied again in different studies; both indicated increased body weight and increased liver glucose at hatch (55, 115, 132).

Al-Murrani (133) first experimented with supplementation of amino acids to the yolk sac at the 7th day of incubation. Results indicated that the embryo did not use the protein until late-stage embryonic development to gain weight and they carried the additional weight through market age. Ohta et al. (117) injected amino acids into the yolk sac at both 0th and 7th days; both injections resulted in increased body weight with no effect on hatchability. More recently, Ohta and Kidd (134) injected amino acids into eggs and observed synonymous results with increased body weight at hatch, when injections were administered to the yolk or the extra-embryonic celom. Ohta et al. (135) dispensed amino acids to the yolk sac at 7 days of incubation, which resulted in increased amino acid concentrations. Other studies have observed that late-term embryonic mortality had been significantly reduced and hatchability was increased by the injection of amino acids (136, 137). Both Ohta et al. (117) and Ohta and Kidd (134) concluded that the addition of amino acids stimulated the utilization and synthesis of amino acids with a simultaneous decrease in the degradation of amino acids (exact biochemical degradation not specified), when the amino acids injected were identical to those naturally occurring in the egg. In summary, the experimental studies of injections with amino acids and proteins indicate the potential benefits for the commercialization of *in ovo* injection of nutrients.

## Immunostimulants

There has been interest in identifying substances that may enhance the development or response of the immune system at an earlier age. Because modern chickens reach market weight at a mere 6–8 weeks, the first days of the chicks’ life account for a larger portion of their lifespan [comparatively to decades past; (9)]. In this time, they are exposed to a new environment; therefore, improving the immune response of these immature chicks is crucial for survival and performance to market age (138). Additionally, stimulating the immune system and taking prophylactic measures rather than having to use therapeutic dosages is superior from a food safety and public health viewpoint (139). Some experimentation to improve immunocompetence *via in ovo* injection of vitamins, amino acids, and carbohydrates has been attempted; results have suggested their beneficial influence on antibody and macrophage response, immunomodulation, and humoral and cellular immunity (128, 131, 140, 141). Additionally, injections of antibodies and antibiotics have been attempted (126, 127). Increased antibody residues in both the yolk sac and blood serum were observed as a result of *in ovo* injection of antibiotics on the 18th day into the amnion. This was associated with reduced establishment of a competitive exclusion culture when embryonated eggs were supplied with PREEMPT™ [commercial competitive exclusion culture isolated from cecal microbiota of healthy adult chickens; (132)].

CpG motifs are able to serve as effective stimulators of the immune system by being recognized as non-self DNA and, thus, promoting host immune response. CpG-ODN has been an effective immunostimulant in mature chickens against *Escherichia coli*, which led to experimentation with delivery *via in ovo* injection (139). Taghavi et al. (129) injected CpG-ODN *in ovo* at 18 days to the amniotic fluid of embryonated eggs and subsequently challenged with *Salmonella* Typhimurium (2 days post-hatch). Results indicated a significantly lower rate of infection and septicemia caused by *S*. Typhimurium (129).

## Altering the *In ovo* Gut Microbiome

### Live Bacterial Strains: Probiotic and Competitive Exclusion

Research regarding the *in ovo* injection of either probiotic bacterial strains or competitive exclusion culture is limited. The beneficial effects of probiotics in animal hosts and their underlying mechanisms have been the subject of numerous reviews (24, 142–145). Probiotic bacterial strains work to compete for attachment sites on the intestinal epithelia, utilizing substrates to produce short-chain fatty acids (SCFAs) and other antimicrobial metabolites and stimulating the host’s immune response (142–144, 146). It was the work of Nurmi and Rantala (22) as well as Rantala and Nurmi (147) who indicated that the application of a single probiotic bacterial strain (*Lactobacillus*) did not confer protection against *Salmonella* infection and that the infection was occurring within the first week post-hatch. The work shifted into evaluating the protection provided by inoculation of newly hatched chicks with the intestinal microbiome of adult chickens showing resistance to infection with *Salmonella* (22, 147). This work was successful and brought about the concept of “competitive exclusion.” While there have been several hypotheses for the mechanism of competitive exclusion, four main mechanisms are acknowledged: (1) an unfavorable environment for invading bacteria is created, (2) receptor sites are utilized by commensal bacteria, leaving no space for invading bacteria, (3) antimicrobial substances are produced, and (4) competition for essential nutrients resulting in selection of certain bacterial strains (145, 148, 149).

As aforementioned, the infection of chickens is primarily observed to occur within the first week post-hatch (22, 147). It has been suggested that distribution of competitive exclusion cultures to chicks on the day of hatch results in accelerated maturation of the chick intestinal microbiome, allowing heightened protection only when given prior to exposure to pathogenic bacteria (150, 151). Therefore, inoculation of chicks with probiotic bacterial strains or competitive exclusion cultures at the earliest possible time would likely be the most effective in providing protection against pathogenic bacteria (149). *In ovo* injection with probiotic bacterial strains or competitive exclusion culture would allow the chicks to be equipped with a fully colonized GIT before arriving at the growout houses, where they are likely to be exposed to pathogenic bacteria (similar to the rationale for *in ovo* injection with MD vaccine). Additionally, administration of probiotic bacterial strains to young chicks have been associated with improved growth performances, both body weight and feed conversion ratio (152–154). The *in ovo* distribution of probiotic bacterial strains or competitive exclusion cultures has the potential to improve growth performance as well as improve the protection of chickens from invading pathogens (149).

The results obtained from the research with *in ovo* injection of probiotics and competitive exclusion cultures are variable. Initial experiments were conducted by *in ovo* injection of different dilutions of a competitive exclusion culture into the air cell or just beneath the inner membrane at 18 days of incubation (155). Results indicated that *in ovo* injection with competitive exclusion cultures may be feasible once proper dilutions are experimentally determined (155). Pedroso et al. (156) bolstered the evidence of the benefits of inoculating embryonic chicks with probiotic competitive exclusion. It was found that not only were the embryonic chicks receiving *in ovo* injection displayed increased microbiota diversity but also that these chicks revealed decreased Enterobacteriaceae, the family to which several enteropathogenic bacteria belong, including *Salmonella* spp. and *E. coli* (156). Similarly, aside from determining *Lactobacillus reuteri* as a safe organism to use as a competitive exclusion agent (18 days of incubation, and injections to both the air cell and amniotic fluid had no significant effect on hatchability when compared to control hatch rate), these experiments indicated improved protection from challenges with enteric pathogens. Additionally, improved growth performance was observed when *L. reuteri* was continued in post-hatch feed [in addition to the *in ovo* injections; (141)]. Conversely, the inoculation of eggs with *Lactobacillus acidophilus, Lactobacillus fermentum*, and *Lactobacillus salivarius via in ovo* injection at 18 days of incubation into the air cell indicated no protection when challenged with *Salmonella* (157).

From the previous discussion, it is apparent that *in ovo* injection of various probiotic and competitive exclusion strains may be of value to the poultry industry. However, limitations in the current understanding of appropriate sites and ages for distribution of probiotic bacterial strains and competitive exclusion culture *in ovo* prevent its utilization in the industry. It is necessary to understand the systematic evaluation of bacterial strains that are safe to use, the dosages, and the age and site of injection before this technique can be practically beneficial to the poultry industry (122).

Limitations of *in ovo* injection with probiotic bacterial strains and competitive exclusion cultures exist and remain to be reconciled. The GIT microbiome is a niche for bacteria of all sorts – beneficial, harmful, and toxic – to live in concert with one another (123). The diversity of the population allows the microbiome to be protective, efficient, and promote health for the host. Injection with only one or a mixture containing only a few beneficial bacteria will likely not be effective as it is not reflective of a diverse intestinal microbiome of healthy adult chickens. It may not provide the intended protection or health for the host. Additionally, *in ovo* injection with a competitive exclusion culture can introduce unknown species of bacteria (123). Potentially harmful and toxin-producing bacteria that are detrimental to the health of the embryonated egg, rather than being beneficial, may be introduced.

### Prebiotics and Synbiotics

Experimentation into the *in ovo* injection of prebiotics or synbiotics is fairly recent; thus, available research is limited. The rationale of distributing prebiotics and synbiotics to a developing embryo is driven by the recognition that activities of both substances work toward improving GIT health. The definition of prebiotics is variable depending on the source (158). Roberfroid et al. (159) stated that prebiotics contribute to “the selective stimulation of growth and/or activity(ies) of one or a limited number of microbial genus(era)/species in the gut microbiota that confer(s) health benefits to the host.” Hutkins et al. (158) identified that amendments and specifications need to be established, expanding on this concept for clarification in the various industries it affects (health care, food, science, and regulatory). Regardless of the requirements for a substance to be considered a prebiotic, several food ingredients are considered to stimulate growth and/or activity of the intestinal microbiota, resulting in improved host health (159–161). Synbiotics, containing a combination of both prebiotics and probiotics, have also been reviewed and discussed based on whether the contained prebiotic is specific to the probiotic contained in the mixture or if it is stimulatory to any intestinal bacterial strains (6). Nevertheless, prebiotics and synbiotics have been attempted for *in ovo* injection, equipping developing embryos to better protect themselves from the various exposures they may encounter upon hatching (123–125).

Distribution *via in ovo* injection of probiotic bacterial strains with prebiotics at 12 days of incubation to the air cell demonstrated little influence on carcass weight and pectoral muscle percentage (124). Similarly, when evaluating prebiotics (inulin) and synbiotics (inulin with *Lactococcus lactis*; validated composition of mixtures in previous *in vitro* experiments with animal models), it was observed that *in ovo* injection into the air cell at 12 days did not impact the feed conversion ratio, yet distribution with prebiotics alone significantly increased the final body weight. Additionally, the delivery of both synbiotics and prebiotics increased the activities of the pancreatic enzymes amylase, lipase, and hydrolase (125). These enzymes are involved in the digestion of food; thus, it is probable that the increased activity is beneficial to the newly hatched chicks in their transition from endogenous to exogenous nutrients (125).

Considerable attention has been given to the effect on immune response and activity when prebiotics or synbiotics are delivered *in ovo*. Madej and Bednarczyk (162) observed *in ovo* injection of synbiotics to be more stimulatory of gut-associated lymphoid tissue (GALT, includes Peyer’s patches, cecal tonsils, Meckel’s diverticulum, and esophageal and pyloric tonsils) colonization by T cells than injection with prebiotics alone. Further, the *in ovo* injection of synbiotics to the air cell at 12 days was shown to stimulate the development of immune organs (bursa of Fabricius and spleen) as well as increase proliferation of lymphocytes in the thymus (163). Stimulation of synthesis of immunoglobulins has also been demonstrated with the *in ovo* distribution of prebiotics and synbiotics at 12 days of incubation to the air cell (164).

Research conducted evaluating the dosages of prebiotic preparations injected *in ovo* demonstrated the current limitations of *in ovo* prebiotic delivery. Villaluenga et al. (123) observed increased numbers of bifidobacteria associated with increased dosage of prebiotic mixtures (various oligosaccharides). However, increased dosages were also negatively associated with hatchability and embryo weight (123). In addition to dosage effects, unknown impacts of *in ovo* prebiotic injection, both the profiles of microorganisms comprising the intestinal microbiome and the development of the chicks post-hatch, need to be investigated (123).

## Conclusion and Future Research

As has been researched thoroughly, the delayed feeding of newly hatched chicks that occurs during the transport of chicks to the broiler farm has revealed a detrimental impact on the GIT development (10). In addition to the GIT development being stunted, the colonization of microorganisms in the GIT may be hindered as well. Recent research has suggested that the establishment of the GIT microbiome in chick embryos has the potential of developing as early as 16 days of incubation based on the colonized yolk sac (83), while sequencing of the 16S RNA revealed 1-day-old chicks to have diversely colonized cecal contents (21). Therefore, in order to maximize productivity in poultry production, the management of chicks needs to start while the chick is malleable and the environmental factors are controlled. Similar to other animals, the best time appears to be when the chick is still developing as an embryo *in ovo*. It is evident that the administration of substances may have positive effects on growth performance and prevention of pathogen invasion (Table 1). There is considerable research to be conducted in order to evaluate how lucrative the *in ovo* administration of various biologics may be. As is evident by the variable results of experiments with differing substances (excluding vaccinations), injection sites, and injection times, there needs to be a standardized method of injection for each substance or groups of substances (for example, carbohydrates, proteins, and probiotic bacterial strains). However, the commercial potential of *in ovo* administration is apparent.

In addition to determining specifications for injectable substrates, further research identifying exactly how receptive a chick embryo is needs to be evaluated. There is reason to believe that the chick embryo GIT is already beginning to be colonized with microorganisms (83). *In ovo* injection with probiotic bacterial strains as well as competitive exclusion culture may prove to be protective against early exposure to pathogens when the chick is most vulnerable. This is similar to what has been observed in the MD vaccine: the earlier the vaccination, the longer time the chick has to develop appropriate immune responses (97). This idea of establishing the intestinal microbiome by *in ovo* injection of beneficial bacteria may be influential on the overall health and well-being of the poultry host.

## Author Contributions

All authors conceived and participated actively in the revisions of the manuscript. SMR wrote the manuscript; YK and SCR provided additional sources of literature references. All authors approved the final submission version of the manuscript and agreed to be held accountable for the content herein.

## Conflict of Interest Statement

The authors declare that the review of the literature was conducted in the absence of any commercial or financial relationships that could be construed as a potential conflict of interest.
